# Virtual MR elastography and continuous-time random walk for predicting Ki-67 status in cervical cancer: a preliminary study

**DOI:** 10.3389/fonc.2026.1843126

**Published:** 2026-08-18

**Authors:** Hongxia Wang, Gaiyun Zhang, Shuaina Wang, Yan Kong, Wangyi Liu, Yaqi Zang, Dongming Han

**Affiliations:** 1 Department of MRI, The First Affiliated Hospital of Henan Medical University, Weihui, China; 2 Department of Ultrasound, The First Affiliated Hospital of Henan Medical University, Weihui, China

**Keywords:** cervical cancer, continuous-time random walk, Ki-67, magnetic resonance imaging, virtual magnetic resonance elastography

## Abstract

**Background:**

This study aimed to evaluate the performance of virtual magnetic resonance elastography (vMRE) and continuous-time random walk (CTRW) models in predicting the Ki-67 status of cervical cancer (CC).

**Methods:**

A total of 85 CC patients were enrolled. Moreover, parameters derived from CTRW (β, α, and D_m_) and diffusion-weighted imaging (DWI) were calculated and compared between higher- and lower-proliferative groups. Moreover, logistic regression (LR) analysis was utilized to find independent predictive factors and to develop a combined diagnostic model. Diagnostic efficacy was assessed utilizing the DeLong test, AUC, and calibration curves.

**Results:**

The μ value was substantially greater in the high-proliferative group than in the low-proliferative group, whereas α, β, D_m_, and ADC values were notably lower in the high-proliferative group. Moreover, the respective AUCs were 0.875 (95% CI:0.785 ~ 0.937), 0.860 (95% CI:0.768 ~ 0.926), 0.815 (95% CI:0.716 ~ 0.891), 0.789 (95% CI:0.687 ~ 0.870), and 0.769 (95% CI:0.668 ~ 0.854). LR analysis confirmed μ, α, D_m_, and ADC as independent predictors of Ki-67 status in CC. The combined diagnostic model incorporating these predictors achieved the highest performance (AUC = 0.970, 95% CI:0.908 ~ 0.995), significantly outperforming individual modalities such as vMRE (μ), DWI (ADC), and single parameters α, β, and D_m_ (Z = 2.891, 4.271, 2.692, 3.102, and 4.064, respectively; all P < 0.05). Furthermore, calibration curves demonstrated favorable stability for the combined diagnostic model.

**Conclusion:**

Both vMRE and CTRW show better performance than conventional DWI in differentiating high- from low-proliferative CC. The combined use of μ, α, Dm, and ADC provides preliminary, hypothesis-generating evidence as a potential imaging biomarker for evaluating Ki-67 status, which requires further validation in larger independent cohorts.

## Introduction

Cervical cancer (CC) is one of the most frequently diagnosed malignancy and cancer-related mortality’s leading cause among women around the globe (1). Its incidence has been steadily increasing, particularly in developing countries and regions, with a notable rise in cases among younger populations (2). Ki-67, a nuclear protein critical for sustaining chromatin integrity during cellular division, is widely recognized as a critical biomarker for evaluating tumor cell proliferation and malignant potential (3). Extensive evidence has linked Ki-67 overexpression to poor clinical outcomes and accelerated disease progression in CC (4, 5). Although tissue biopsy is still considered to be the gold standard for assessing proliferative activity, its invasive nature, along with variability stemming from sampling limitations and technical inconsistencies, can compromise the reproducibility and accuracy of diagnostic evaluations (6). Therefore, the development of non-invasive, reliable methods to determine Ki-67 status is of great importance for advancing personalized treatment strategies in CC patients.

The development of quantitative MRI has provided a valuable non-invasive technique for assessing Ki-67 status in tumors. Diffusion-weighted imaging (DWI), a broadly utilized quantitative MRI technique in clinical practice, produces the apparent diffusion coefficient (ADC) as a key parameter that replicates the diffusion behavior of water molecules within biological tissues (7). Numerous studies have demonstrated the utility of ADC in estimating Ki-67 status across various malignancies, including soft tissue sarcoma (8), astrocytoma (9), and CC (10). However, conventional DWI is based on a mono-exponential model that assumes Gaussian water diffusion, which oversimplifies the complex microstructural environment of tissues and limits its accuracy in capturing true diffusion characteristics (11). Consequently, the diagnostic performance of standard DWI remains suboptimal. Virtual magnetic resonance elastography (vMRE) has recently emerged as a novel quantitative MRI technique derived from DWI (12). Unlike conventional magnetic resonance elastography (MRE), which requires external mechanical excitation and specialized hardware, vMRE noninvasively quantifies tissue stiffness without such additional requirements (13). Recent clinical studies have demonstrated its utility in predicting histological grades of rectal cancer (14) and differentiating benign from malignant pulmonary nodules (15). Continuous-time random walk (CTRW) represents another advancement in DWI modeling. This approach employs a non-Gaussian distribution as its foundational framework, enabling a more comprehensive representation of the complex diffusion behavior of water molecules within tissues and thus providing a more accurate assessment of lesions (16). Recent studies have applied CTRW in the assessment of glioma (17), breast cancer (18), and prostate cancer (19). However, in the context of CC, only a inadequate number of investigations have researched the value of CTRW (20, 21), and to our knowledge, no authoritative reports have yet been published regarding vMRE.

Therefore, this study aims to collect multimodal MRI data, including vMRE and CTRW sequences, from patients with CC. Additionally, it seeks to compare and evaluate the effectiveness of relevant quantitative parameters in distinguishing between high- and low-proliferative CC. Ultimately, the goal is to establish novel imaging biomarkers that enable the non-invasive assessment of Ki-67 status in CC.

## Materials and methods

### Participants

This study was conducted per the Declaration of Helsinki and got approval from the local institutional review board. Moreover, written informed consent was taken from each patient or their legal representative following a thorough explanation of the examination-related procedures and precautions prior to pelvic MRI scanning. A consecutive cohort of 115 patients with clinically suspected CC was recruited for pelvic MRI evaluation between May 2021 and November 2025. Patients were excluded following the criteria: (i) incomplete acquisition of the full MRI protocol (n = 6); (ii) prior antitumor treatment (n = 7); (iii) histopathological diagnoses other than cervical cancer or indeterminate findings (n = 8); (iv) poor image quality precluding definitive diagnostic interpretation (n = 6); and (v) an interval greater than two weeks between MRI scanning and histopathological biopsy (n = 3). After applying these exclusion criteria, 85 patients remained in the final study cohort. Demographic and clinical data, including age, maximum tumor diameter, pathological subtype, and International Federation of Gynecology and Obstetrics (FIGO) stage, were prospectively collected for subsequent statistical analysis.

### Scanning protocol of MRI

MRI examinations were carried out utilizing a 3.0T Signa Architect scanner (GE Healthcare, USA) fortified with a 16-element phased-array torso coil. Participants were positioned supine and entered the scanner feet-first. Initially, axial T2-weighted imaging (T2WI) was obtained to localize the tumor, using the parameters as follows: slice thickness = 6 mm; repetition time/echo time (TR/TE) = 2942/122.24 ms; slice gap = 1 mm; field of view (FOV) = 256 × 80 mm; number of excitations (NEX) = 1; and matrix size = 320 × 256. The acquisition time for this sequence was 1 minute and 35 seconds. Guided by the T2WI images, multi-b-value diffusion sequences were then obtained for slices containing the lesion, maintaining consistent positioning, slice thickness, and gap settings. The diffusion sequence parameters were: b-values = 0, 50, 80, 100, 150, 200, 300, 500, 800, 1000, 1300, 1500, 1800, 2400, 3000, and 3600 s/mm²; corresponding NEX values of 1, 1, 1, 1, 1, 1, 1, 1, 2, 2, 2, 4, 4, 6, 6, and 6; TR/TE = 2011/72.3 ms; FOV = 256 × 80 mm; and matrix size = 128 × 128. This sequence’s total acquisition time was 9 minutes and 12 seconds.

### Data processing

All data processing was done utilizing MATLAB R2018b (MathWorks Inc., Natick, MA, USA). Firstly, the multi-b-value sequence datasets underwent preprocessing to correct for B_0_ field distortion, gradient nonlinearity, and eddy current-induced artifacts. All b-value volumes were aligned to the same spatial reference frame during this step to ensure inherent spatial consistency across subsequent parametric maps. Following preprocessing, pseudocolor parameter maps for DWI, vMRE, and CTRW models were generated based on Equations 1–3, respectively.

(1) $${\text{S}}_{\text{b}}/{\text{S}}_{0}=\text{exp}(-\text{b}\times \text{ADC})$$

In the DWI equation, b indicates the diffusion sensitizing factor. The signals acquired at b-values of 0 to 800 s/mm² are designated as S_0_ and S_b_, respectively.

(2) $$\mu_{MRE}=\alpha \cdot In\left(S_{low}/S_{high}\right)+\beta$$

In the vMRE equation, S_low_ and S_high_ indicate the signal intensities acquired at b-values of 200 s/mm² and 1500 s/mm², respectively (22). The α (scaling factor) and β (shift factor), based on previous studies, were independently set to −9.8 ± 0.8 and 14.0 ± 0.9, respectively (12). Notably, these scaling and shift factors have not been specifically calibrated for CC tissue. The derived μ value serves as a relative quantitative indicator reflecting diffusion-associated tissue biomechanical properties, rather than an absolute measurement of tissue shear modulus.

(3) $$S/S_{0}=E_{\alpha }\left(-{\left(bD_{m}\right)}^{\beta }\right)$$

In the CTRW equation, Ea denotes the Mittag–Leffler function. Maps of α (temporal diffusion heterogeneity coefficient), β (spatial diffusion heterogeneity coefficient), and D_m_ (anomalous diffusion coefficient) were generated through nonlinear fitting of voxel-wise signal intensities from multi-b-value data. During subdiffusion in heterogeneous lesion microenvironments, the dimensionless parameters α and β typically range between 0 and 1 (17).

### Parameter computation​

The entire tumor volume was manually segmented utilizing ITK-SNAP software (v3.8.0; www.itksnap.org). Segmentation was performed on all axial slices covering the full extent of the tumor to achieve whole-volume analysis. Tumor boundaries were delineated on b = 800 s/mm² DWI sequences by defining regions of interest (ROIs) along the peripheral margins of the lesions. T2WI images were cross-referenced to systematically exclude obvious intratumoral cystic or necrotic areas, hemorrhagic foci, and calcifications. After volumetric segmentation on DWI, the tumor ROIs were transferred automatically to the parametric maps to facilitate calculation of ADC, μ, α, β, and D_m_. Each transferred ROI was manually inspected slice-by-slice on all parametric maps. All segmentation and measurements were independently conducted by two radiologists: an attending radiologist with eight years of experience in abdominal MRI and an associate chief radiologist with eleven years of expertise in gynecological oncology imaging. Both radiologists were blinded to the patients’ clinicopathological information.

### Ki-67 analysis

All formalin-fixed, paraffin-embedded CC specimens underwent hematoxylin- eosin (H&E) staining first for histological confirmation. Immunohistochemical staining for Ki-67 was performed using a mouse anti-human monoclonal antibody (clone MIB-1, DAKO, Glostrup, Denmark) at a 1:100 dilution on a Dako Autostainer Link 48 platform (DAKO, Glostrup, Denmark). For antigen retrieval, slides were incubated in ethylene diaminetetraacetic acid (EDTA) buffer (pH 9.0) and subjected to high-pressure heat treatment for 20 minutes. The EnVision FLEX detection system (DAKO, Glostrup, Denmark) with 3,3’-diaminobenzidine as the chromogen was applied for signal visualization. For each batch of staining, known Ki-67–positive human tonsil tissue was used as a positive control, and phosphate-buffered saline substituted for the primary antibody was used as a negative control to ensure staining quality. Ki-67 status was assessed by a board-certified pathologist with 10 years of experience in gynecological pathology, respectively, who was blinded to all MRI findings and clinical data of the patients. For each specimen, five representative high-power fields (×400 magnification) with the highest density of positive cells (hotspots) were selected under light microscopy. Within each hotspot, 100 tumor cells were counted, and the percentage of Ki-67–positive nuclei was recorded. To stratify patients according to tumor proliferative activity, a cutoff value of 50% was adopted for the Ki-67 labeling index in this study. This threshold was selected because it is the most commonly used stratification criterion in existing imaging and pathological studies of CC, enabling comparability with the majority of published findings in the field (10, 21). However, the 50% threshold has not been uniformly standardized by clinical guidelines, and the optimal cutoff for Ki-67 stratification in cervical cancer remains to be further validated.

### Data analysis

All statistical analyses were conducted utilizing MedCalc software (version 15.0; MedCalc Software, Belgium). Inter-observer reliability of quantitative parameters derived from DWI, vMRE, and CTRW by two radiologists was assessed utilizing the intraclass correlation coefficient (ICC), with values higher than 0.75 suggesting excellent agreement (23). Depending on data distribution, comparisons between proliferation-based groups were done utilizing the independent samples t-test, Mann-Whitney U test, and Chi-square test for normally distributed, non-normally distributed, and categorical variables, respectively. Logistic regression (LR) analysis was applied to find independent predictive factors and to develop a combined diagnostic model. The variance inflation factor (VIF) is used to assess multicollinearity; a value of VIF less than 5 is considered to indicate the absence of multicollinearity. Discriminatory performance was evaluated utilizing the area under the receiver operating characteristic curve (AUC), with AUC comparisons made via the DeLong test. The predictors included in the multivariate model were pre-specified based on prior univariate analysis, with no additional data-driven variable selection performed during model fitting. Internal validation was performed using the bootstrap method with 1,000 resamples. For each bootstrap resample drawn with replacement from the original cohort, the multivariate logistic regression model was fully refitted to re-estimate all regression coefficients. The refitted model was then applied to the original full dataset to obtain test-set performance. The mean optimism was calculated as the average difference between the training-set (bootstrap sample) performance and the test-set (original dataset) performance across all 1000 resamples. The optimism-corrected performance was derived by subtracting the mean optimism from the apparent performance calculated on the original full dataset. Calibration curves were plotted to visualize the agreement between predicted probabilities and observed frequencies; the calibration slope and calibration intercept were calculated to quantify the degree of probability shrinkage and calibration-in-the-large, respectively; the raw Brier score (range 0–1, lower values indicate better overall prediction accuracy) was calculated as a comprehensive measure of prediction performance; and the Hosmer-Lemeshow goodness-of-fit test was performed as a formal statistical test of calibration. A two-sided P-value < 0.05 was deemed statistically significant.

## Results

### Demographic and clinical characteristics

A total of 85 patients with CC were included and stratified according to Ki-67 status into a high-proliferative group (n = 45) and a low-proliferative group (n = 40). Comparative analyses revealed no statistically substantial differences between the 2 groups with respect to demographic and clinicopathological characteristics, including age, maximum tumor diameter, pathological subtype, and FIGO stage (all P > 0.05; **Table 1**).

**Table 1:** Comparison of clinical and imaging variables between high- and low-proliferative groups.

Variables	High-proliferative (n = 45)	Low-proliferative (n = 40)	z/χ^2/^t value	P-value
Clinical				
Age (years)	57.00 (39.00, 64.50)	53.50 (47.25, 59.75)	-0.093	0.926 ^a^
Maximum diameter (mm)	38.10 (20.15, 51.45)	26.25 (16.68, 43.60)	-1.519	0.129 ^a^
Subtype (%)			3.772	0.054 ^b^
SCC	40/45 (89.58%)	29/40 (76.32%)		
AC	5/45 (10.42%)	11/40 (23.68%)		
FIGO Stage (%)			1.377	0.241 ^b^
I-II	35/45 (77.78%)	35/40 (87.50%)		
III-IV	10/45 (22.22%)	5/40 (12.50%)		
Imaging				
α	0.53 (0.50, 0.63)	0.78 (0.66, 0.87)	-5.705	< 0.001 ^b^
B	0.59 (0.55, 0.63)	0.68 (0.63, 0.75)	-4.992	< 0.001 ^b^
D_m_ (×10^−3^mm^2^/s)	0.93 (0.79, 1.05)	1.15 (1.00, 1.45)	-4.587	< 0.001 ^b^
ADC (×10^−3^mm^2^/s)	1.09 ± 0.22	1.35 ± 0.25	-5.144	< 0.001 ^c^
*μ	6.86 ± 1.44	4.85 ± 1.09	7.212	< 0.001 ^c^

SCC, Squamous cell carcinoma; AC, Adenocarcinoma; FIGO, Federation of Gynecology and Obstetrics. The a, b and c correspond to comparisons made by Mann-Whitney U test, chi-squared test, and independent t-test, respectively. *μ is a vMRE-derived relative biomechanical index, not an absolute shear modulus measurement with kPa units.

### ICC evaluation

Interobserver reliability between the two radiologists for ADC, μ, α, β, and Dm was excellent, with all ICC values exceeding 0.75 (**Table 2**). Consequently, subsequent statistical analyses were performed using the averaged measurements from the two observers.

**Table 2:** ICC assessment of each parameter.

Variables	ICC (95% CI)
α	0.917 (0.872 ~ 0.946)
B	0.912 (0.864 ~ 0.943)
D_m_ (×10^−3^mm^2^/s)	0.934 (0.899 ~ 0.957)
ADC (×10^−3^mm^2^/s)	0.946 (0.791 ~ 0.977)
*μ	0.944 (0.914 ~ 0.964)

ICC, intraclass correlation coefficient; 95% CI, 95% confidence interval.

### Parameter comparison

**Figure 1:**
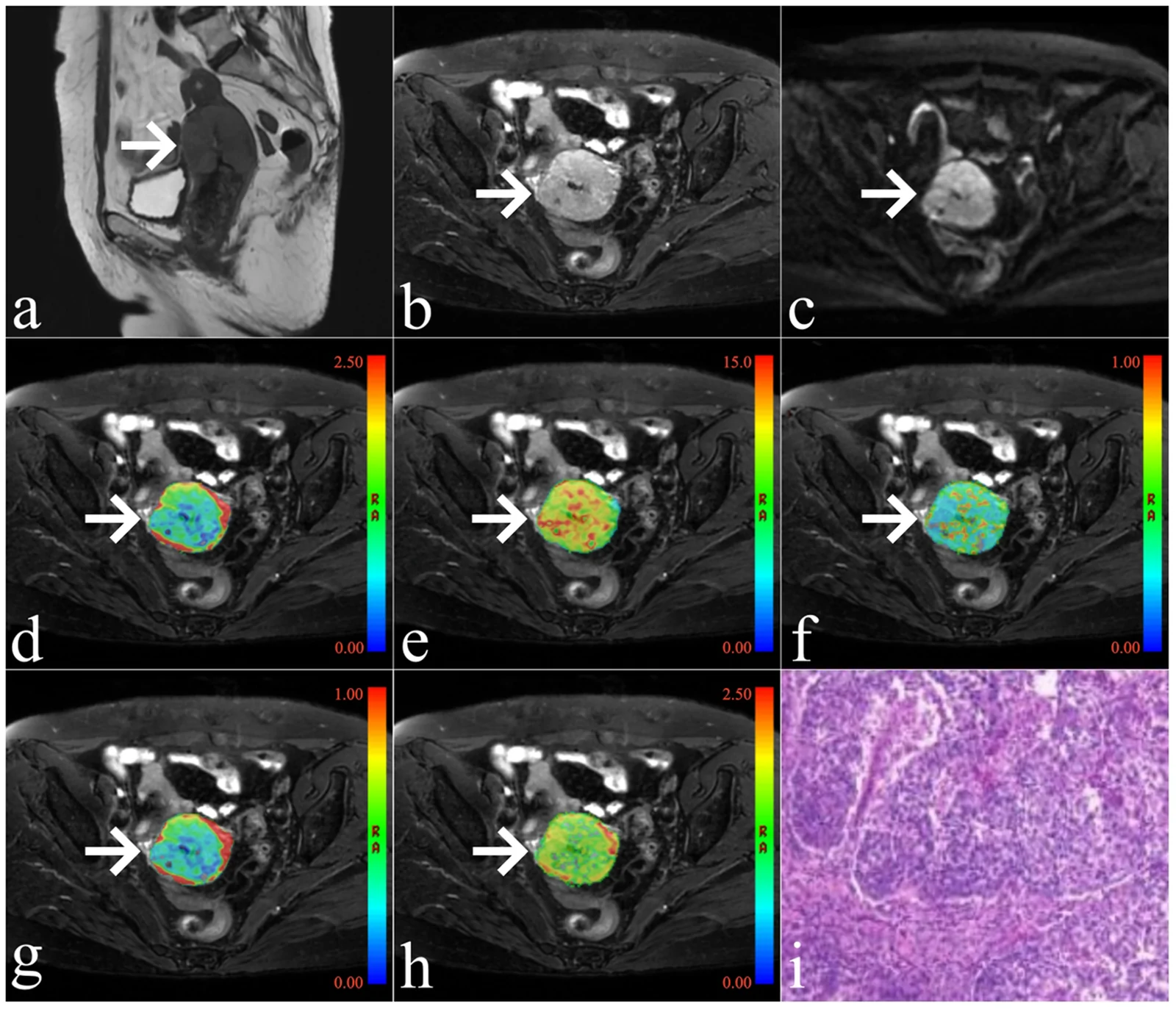
A 72-year-old woman with CC (white arrow, Ki-67 = 70%). **(a)** sagittal T2- weighted image without fat suppression; **(b)** axial fat-suppressed T2-weighted image; **(c)** axial DWI map (b = 800 s/mm^2^); **(d)** axial pseudo-color map of ADC; **(e)** axial pseudo-color map of μ; **(f)** axial pseudo-color map of α; **(g)** axial pseudo-color map of β; **(h)** axial pseudo-color map of D_m_; **(i)** H&E-stained histopathological images (original magnification, 100×).

**Figure 2:**
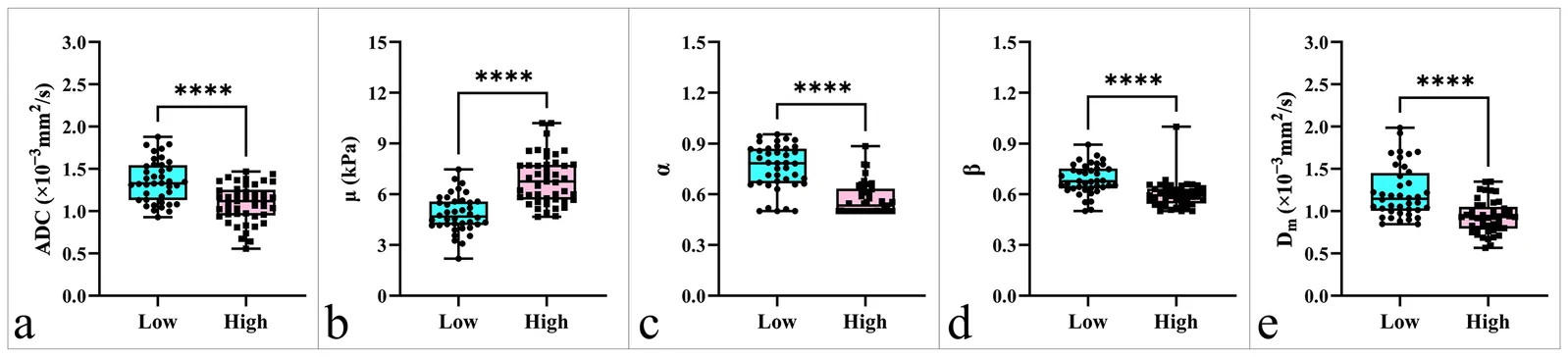
Box plots depicting various parameters in the low- and high-proliferative groups: **(a)** ADC; **(b)** μ; **(c)** α; **(d)** β; and **(e)** D_m_. **** indicates P < 0.0001.

Relative to the lower-proliferative group, the higher-proliferative group demonstrated a substantially higher vMRE-derived relative μ index, whereas α, β, ADC, and Dm values were all significantly lower (all P < 0.05; **Figures 1**, **2**, **Table 1**).

### Independent predictors screening

LR analysis was done to observe the associations between Ki-67 status and a range of variables, including demographic and clinical characteristics (age, maximum tumor diameter, pathological subtype, and FIGO stage) as well as quantitative parameters derived from DWI, vMRE, and CTRW (ADC, μ, α, β, and D_m_). Univariate analysis initially identified ADC, β, μ, α, and D_m_ as significant predictors (all P < 0.05). These variables were subsequently entered into a multivariate model, which confirmed ADC, μ, α, and D_m_ as independent predictors of proliferation status (P = 0.040, 0.020, 0.004, and 0.011, respectively; **Table 3**).

**Table 3:** Univariate and multivariate LR analyses for clinical and imaging variables.

Variables	Univariate analyses		VIF	Multivariate analyses	
	OR* (95% CI)	*P*-value		OR* (95% CI)	*P*-value
Clinical					
Age (years)	1.000 (0.651 ~ 1.534)	0.998	1.023	/	/
Maximum diameter (mm)	1.339 (0.858 ~ 2.089)	0.199	1.542	/	/
Subtype (%)	1.547 (0.980 ~ 2.442)	0.061	1.133	/	/
FIGO Stage (%)	1.304 (0.832 ~ 2.044)	0.246	1.601	/	/
Imaging					
α	0.166 (0.080 ~ 0.342)	< 0.001	1.473	0.115 (0.026 ~ 0.498)	0.004
B	0.258 (0.130 ~ 0.514)	< 0.001	1.246	0.510 (0.178 ~ 1.460)	0.209
D_m_ (×10^−3^mm^2^/s)	0.216 (0.100 ~ 0.466)	< 0.001	1.410	0.129 (0.027 ~ 0.625)	0.011
ADC (×10^−3^mm^2^/s)	0.263 (0.136 ~ 0.507)	< 0.001	1.349	0.303 (0.097 ~ 0.946)	0.040
*μ	9.449 (3.562 ~ 25.065)	< 0.001	1.727	9.247 (1.413 ~ 60.511)	0.020

OR*, odds ratio per 1 standard deviation increment; 95% CI, 95% confidence interval; VIF, variance inflation factor. All factors with a P-value < 0.05 and a VIF < 0.05 in univariate Logistic Regression (LR) analysis were incorporated into subsequent multivariate LR analysis. *μ is a vMRE-derived relative biomechanical index, not an absolute shear modulus measurement with kPa units.

### Diagnostic performance

**Figure 3:**
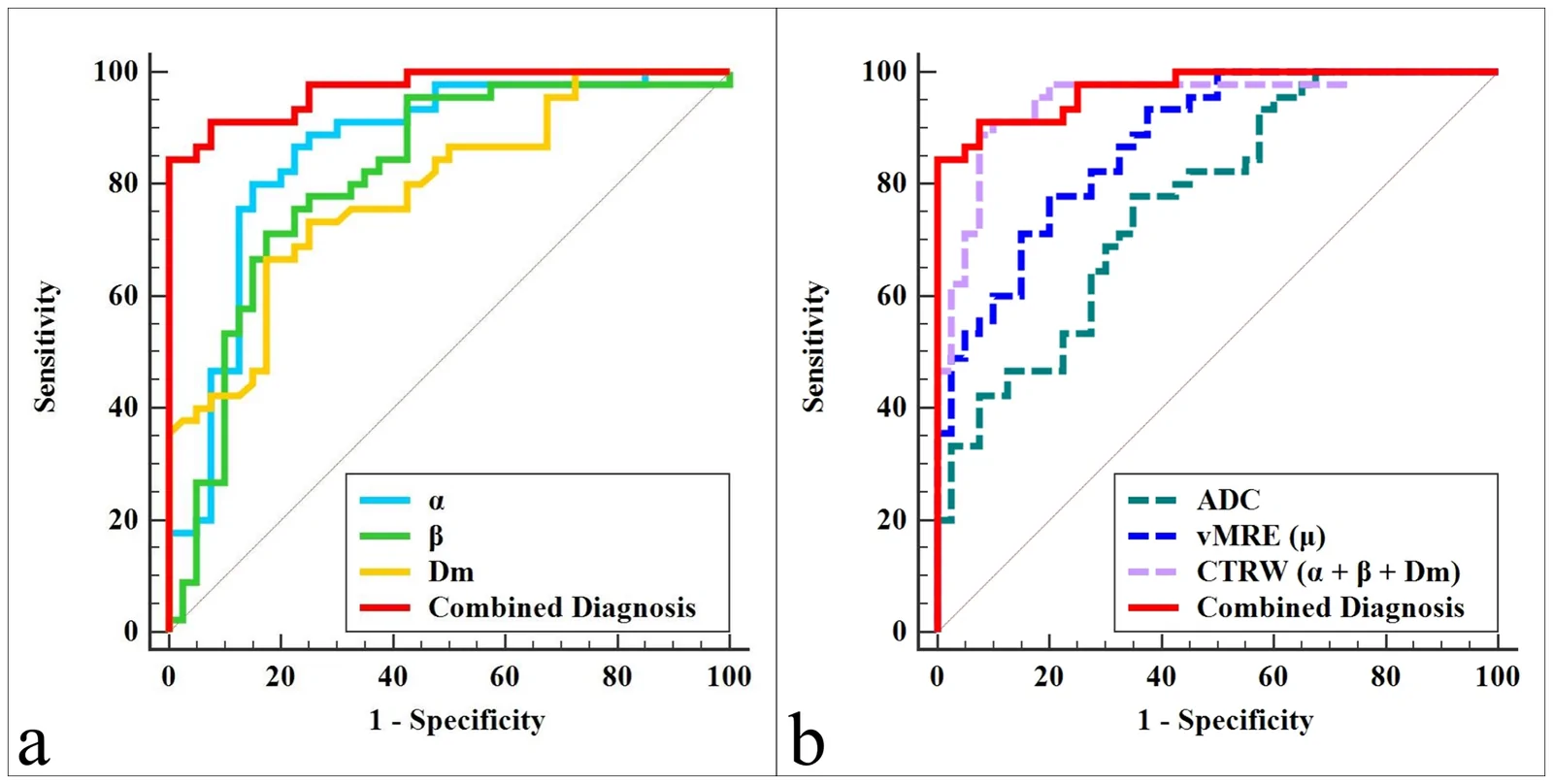
Receiver operating characteristic (ROC) curves for discriminating high proliferative and low-proliferative groups using individual parameters and the combined model of all independent predictors (ADC + μ + α + Dm). **(a)** ROC curves of individual CTRW-derived parameters (α, β, Dm) and the combined diagnostic model. **(b)** ROC curves of ADC, vMRE (μ), CTRW (α + β + Dm), and the combined diagnostic model. The AUC values were listed as follows: ADC = 0.769 (95% CI: 0.668–0.854), vMRE (μ) = 0.875 (95% CI: 0.785–0.937), α = 0.860 (95% CI: 0.768–0.926), Dm = 0.815 (95% CI: 0.716–0.891), β = 0.789 (95% CI: 0.687–0.870), CTRW (α + β + Dm) = 0.948 (95% CI: 0.877–0.984), combined model = 0.970 (95% CI: 0.908–0.995).

The independent predictors’ (ADC, μ, α, and D_m_) combination yielded an AUC of 0.970 (95% CI:0.908 ~ 0.995), with a sensitivity of 84.44% and a specificity of 100.00%, illustrating excellent diagnostic performance. This combined model significantly outperformed individual modalities, including vMRE (μ; AUC = 0.875, 95% CI:0.785 ~ 0.937) and DWI (ADC; AUC = 0.769, 95% CI:0.668 ~ 0.854), as well as individual CTRW-derived parameters such as α (AUC = 0.860, 95% CI:0.768 ~ 0.926), β (AUC = 0.815, 95% CI:0.716 ~ 0.891), and D_m_ (AUC = 0.789, 95% CI:0.687 ~ 0.870). The corresponding Z values were 2.891, 4.064, 4.271, 2.692, and 3.102, respectively (all P < 0.05). Conversely, no statistically substantial variance in AUC was observed between the combined model and the CTRW combination (α + β + D_m_; AUC = 0.948, 95% CI:0.877 ~ 0.984; Z = 1.054; P = 0.292). Detailed results are presented in **Figure 3**, **Table 4**.

**Table 4:** Diagnostic performance comparison and analysis.

Variables	AUC (95% CI)	Specificity	Sensitivity	Cutoff	Comparison with combined diagnosis
α	0.860 (0.768 ~ 0.926)	85.00%	80.00%	0.652	Z = 2.692, P = 0.007
B	0.815 (0.716 ~ 0.891)	82.50%	71.11%	0.620	Z = 3.102, P = 0.002
D_m_	0.789 (0.687 ~ 0.870)	82.50%	66.67%	0.957	Z = 4.064, P < 0.001
DWI/ADC	0.769 (0.668 ~ 0.854)	65.00%	77.78%	1.262	Z = 4.271, P < 0.001
vMRE/μ	0.875 (0.785 ~ 0.937)	80.00%	77.78%	5.629	Z = 2.891, P = 0.004
CTRW	0.948 (0.877 ~ 0.984)	92.50%	88.89%	0.581	Z = 1.054, P = 0.292
Combined Diagnosis	0.970 (0.908 ~ 0.995)	100.00%	84.44%	0.671	/

Combined Diagnosis = α + D_m_ + ADC + μ, CTRW = α + β + D_m_ 95% CI = 95% confidence interval.

### Bootstrap correction and calibration

**Figure 4:**
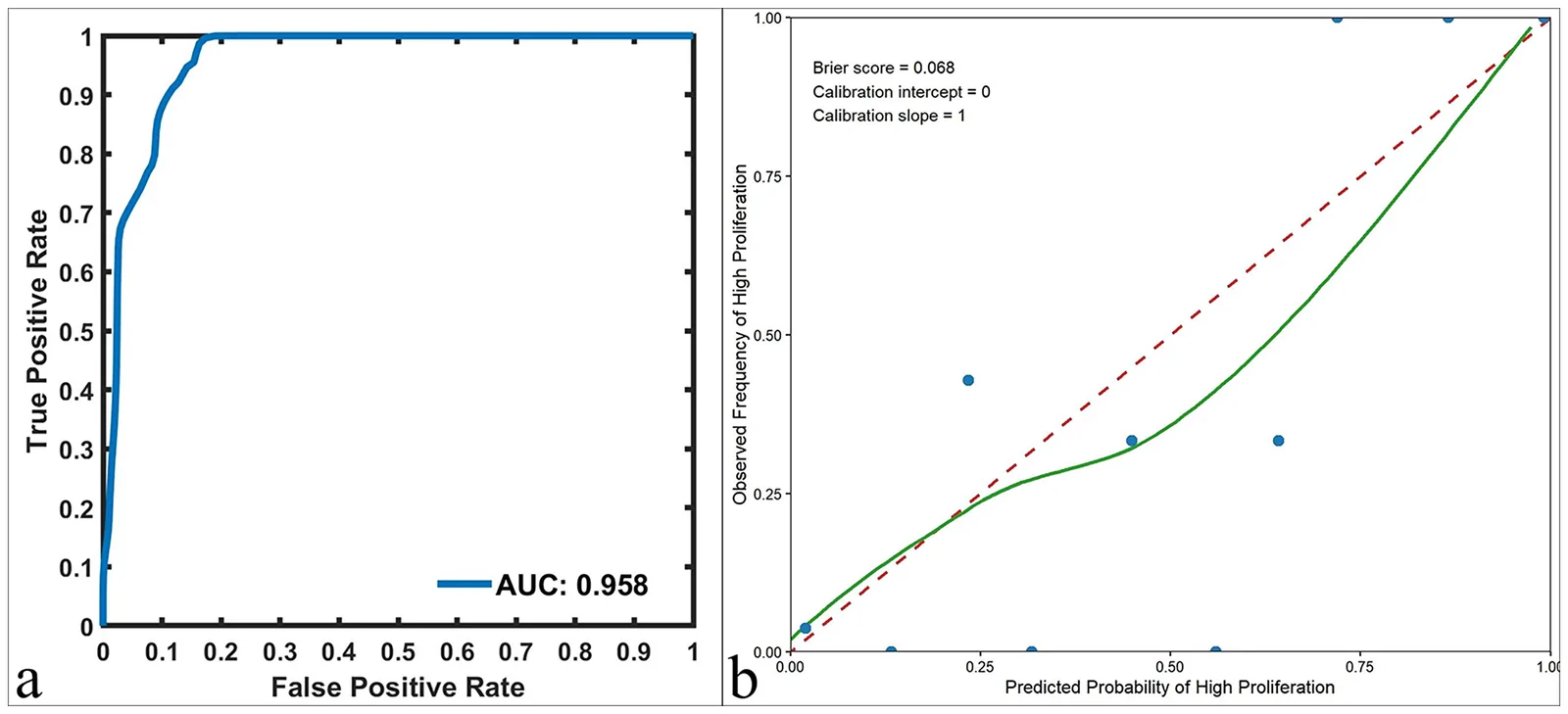
**(a)** ROC curve of the combination of independent predictors (ADC + μ + α + D_m_) verified internally by bootstrap, and the optimism-corrected AUC was 0.958 (95% CI: 0.942–0.966). **(b)** Calibration curve illustrating the agreement between the predicted probability of high proliferation and the actual observed frequency based on the combination of independent predictors (ADC + μ + α + D_m_). The dashed diagonal line represents the ideal calibration reference (perfect agreement between predicted probability and observed frequency). The solid line represents the observed calibration trend fitted by LOESS smoothing. Quantitative calibration metrics: Brier score = 0.068, calibration intercept = 0.000, calibration slope = 1.000.

Internal validation based on 1,000 bootstrap resamples demonstrated high stability of the combined diagnostic model. The optimism-corrected AUC was 0.958 (95% CI: 0.942–0.966), with an estimated mean optimism of only 0.012 (**Figure 4a**). For calibration performance, the model achieved a raw Brier score of 0.068, indicating high overall prediction accuracy. The calibration intercept was 0.000, indicating excellent calibration-in-the-large without systematic bias in global probability estimation, and the calibration slope was 1.000, indicating no significant shrinkage or expansion of predicted probabilities across the full probability range. The Hosmer-Lemeshow goodness-of-fit test showed no statistically significant deviation between predicted and observed values (χ² = 2.159, df = 7, P = 0.951), further confirming good calibration of the model. The calibration curve also showed good agreement between predicted high-proliferation probability and actual observed frequency (**Figure 4b**).

## Discussion

To date, the clinical value of vMRE and CTRW modeling in the evaluation of CC remains incompletely characterized. In this investigation, we illustrated that the DWI-derived parameter ADC, the vMRE-derived parameter μ, and the CTRW-derived parameters α, β, and D_m_ can effectively discriminate between high- and low-proliferative CC. Furthermore, multivariate logistic regression analysis identified ADC, μ, α, and D_m_ as independent predictors of Ki-67 status. The combined application of these parameters achieved optimal diagnostic performance, as evidenced by high AUC values and well-fitted calibration curves, indicating preliminary clinical reliability. Collectively, these preliminary findings provide hypothesis-generating evidence for a multimodal non-invasive imaging biomarker framework for Ki-67 status assessment in cervical cancer, and lay a methodological foundation for subsequent large-scale confirmatory studies.

DWI is a conventional MRI technique utilized to evaluate the diffusion behavior of water molecules in biological tissues under the assumption of a Gaussian distribution (8). The ADC, a key quantitative parameter derived from DWI, serves as a validated indicator of tissue water diffusion capacity (24). Previous research has shown that tumors with high Ki-67 expression typically exhibit greater malignant potential and more compact cellular architecture than those with low Ki-67 expression. As a result, water diffusion in highly proliferative lesions is more limited, resulting in reduced ADC values (9, 10). In line with these findings, our study demonstrated meaningly lower ADC values in the high-proliferative group relative to the lower-proliferative group, supporting the DWI’s utility for evaluating Ki-67 status in CC.

The vMRE represents a novel MRI technique distinct from conventional DWI, enabling the quantitative estimation of tissue shear modulus by calibrating ADC shifts derived from standard MRE principles using only two b-values. This approach provides valuable, non-invasive insights into tissue biomechanical stiffness without the need for external mechanical excitation (12). Yang et al. reported that vMRE effectively differentiates benign from malignant pulmonary tumors (25), while Kromrey et al. demonstrated its ability to distinguish liver fibrosis stages with diagnostic performance comparable to that of conventional MRE (26). Building upon these applications, the present study explored the potential of vMRE for assessing tumor proliferative status associated with Ki-67 expression in CC. Our results revealed significantly higher μ values in the higher-proliferative group than in the lower-proliferative group. We hypothesize that this finding reflects the rapid and disorganized cellular proliferation characteristic of highly malignant CC, which promotes intratumoral stromal deposition and structural remodeling, ultimately increasing tissue biomechanical stiffness as reflected by the relative vMRE μ index (27). Additionally, accelerated tumor growth generates intratumoral mechanical stress, elevating tension within extracellular matrix collagen networks and further enhancing biomechanical stiffness within the tumor microenvironment (28, 29).

The CTRW model characterizes water diffusion in biological tissues by abandoning the classical Gaussian diffusion assumption and adopting a more sophisticated fitting framework that decouples tissue heterogeneity into temporal and spatial dimensions (30). Within this model, D_m_ reflects the overall diffusion rate. In this study, D_m_ values were substantially lower in the high-proliferative group than in the low-proliferative group, likely due to the denser microstructural organization of highly proliferative tumors, which imposes greater restrictions on water diffusion (31). The parameters α and β quantify spatial and temporal diffusion heterogeneity, respectively. In homogeneous tissue environments, both parameters approach 1, indicating near-Gaussian diffusion behavior. However, in microstructurally complex tissues, such as densely packed malignant lesions, α and β typically decrease, reflecting anomalous diffusion characterized by increased temporal and spatial constraints (16, 21). Chang et al. reported a negative relation between β and Ki-67 expression in breast cancer (32), while a small-sample study by Su et al. involving 55 CC patients found lower α values in the Ki-67 high-expression group (21). In line with these reports, our study observed significantly declined β and α values in the high-proliferative group, with α identified as an independent predictor of Ki-67 status. These findings further support the utility of CTRW-derived parameters in CC assessment. Nevertheless, some studies have reported contradictory results (33), and others have suggested limited utility of α and β for Ki-67 evaluation (34). Future investigations with larger sample sizes and standardized CTRW fitting protocols are therefore warranted to improve the robustness and reproducibility of these findings.

Despite its promising results, this study has several limitations. Firstly, the vMRE and CTRW acquisition protocols were adopted from previous studies and were not specifically optimized for CC imaging. For vMRE calculation, general empirical scaling and shift factors were applied without calibration against conventional MRE. Therefore, the μ parameter cannot be interpreted as a direct absolute measurement of cervical cancer tissue stiffness in kPa, and its biophysical meaning as a surrogate of tissue rigidity in the pelvic cervical cancer scenario requires further validation against conventional MRE. Secondly, as a one-center study with a comparatively smaller sample size, there is a risk of overrating diagnostic performance. Third, the multi-b-value diffusion sequence was based on single-shot echo-planar imaging, which led to the exclusion of some small lesions due to insufficient visualization and associated analytical limitations. Fourth, although the 50% cutoff for Ki-67 proliferative status used in this study is widely adopted in current CC research, it has not been uniformly standardized by clinical guidelines. The diagnostic performance of the imaging model may differ when alternative Ki-67 thresholds are applied, and the optimal cutoff value still needs to be explored in larger sample cohorts. Fifth, Ki-67 status assessment was performed by a single senior pathologist, without a second independent blinded reader and corresponding interobserver agreement analysis. Although this pathologist has extensive diagnostic experience in gynecological pathology, the absence of inter-reader reliability testing may introduce potential assessment bias and affect the reproducibility of the reference standard. Accordingly, the findings of this study should be interpreted as hypothesis-generating rather than clinically actionable. In the future, we will further increase the sample size and conduct multicenter studies. At the same time, we will optimize scanning parameters based on the histological characteristics of CC and attempt to compare our results with those of conventional MRE, with the aim of obtaining more consistent and reliable results.

## Conclusion

Both vMRE and CTRW demonstrate superior discriminative performance compared with conventional DWI for distinguishing cervical cancer with high and low proliferative activity. The combined model incorporating ADC, μ, α, and Dm provides preliminary evidence for non-invasive assessment of Ki-67 status in patients with cervical cancer. The current findings are hypothesis-generating in nature, and further large-scale, multicenter external validation is required before any potential clinical translation.

## Data Availability

The raw data supporting the conclusions of this article will be made available by the authors, without undue reservation.
